# Correction to: The impact of demographics on organ damage in Behçet’s syndrome: a cross-sectional analysis of the international PROBE cohort

**DOI:** 10.1093/rheumatology/keaf667

**Published:** 2025-12-30

This is a correction to: Alberto Floris, Riccardo Laconi, Sarra Chadli, Ahmed Laymouna, Mohamed Tharwat Hegazy, Yeliz Yagiz Ozogul, Yesim Ozguler, Luísa Serpa Pinto, Majid Alikhani, Natalia Lurdes Oliveira, Jurgen Sota, Giuseppe Lopalco, Pietro Carboni, Dimitri Poddighe, Nikolaos Kougkas, Alireza Khabbazi, Alexandre Wagner Silva de Souza, Andrea Lo Monaco, Gerard Espinosa, Ronald R Butendieck Jr, Marcello Govoni, Bakytsholpan Issayeva, Florenzo Iannone, Claudia Fabiani, Luca Cantarini, Farhad Shahram, João Araújo Correia, Zoubida Tazi Mezalek, Gaafar Ragab, Laurent Arnaud, Alberto Cauli, PROBE collaborators, Gulen Hatemi, Matteo Piga, The impact of demographics on organ damage in Behçet’s syndrome: a cross-sectional analysis of the international PROBE cohort, *Rheumatology*, 2025, https://doi.org/10.1093/rheumatology/keaf636

The following changes have been made to the originally published manuscript. A co-author’s name is corrected to read: “Zoubida Tazi Mezalek”.

